# Spatiotemporal imaging and pharmacokinetics of fluorescent compounds in zebrafish eleuthero-embryos after different routes of administration

**DOI:** 10.1038/s41598-021-91612-6

**Published:** 2021-06-09

**Authors:** Marlly Guarin, Ruben Faelens, Arianna Giusti, Noémie De Croze, Marc Léonard, Deirdre Cabooter, Pieter Annaert, Peter de Witte, Annelii Ny

**Affiliations:** 1 Laboratory for Molecular Biodiscovery, Department of Pharmaceutical and Pharmacological Sciences, University of Leuven, Leuven, Belgium; 2 L’Oréal, Research and Innovation, Aulnay-sous-Bois, France; 3 Pharmaceutical Analysis, Department of Pharmaceutical and Pharmacological Sciences, University of Leuven, Leuven, Belgium; 4 Drug Delivery and Disposition, Department of Pharmaceutical and Pharmacological Sciences, University of Leuven, Leuven, Belgium

**Keywords:** Computational biology and bioinformatics, Drug discovery

## Abstract

Zebrafish (*Danio rerio*) is increasingly used to assess the pharmacological activity and toxicity of compounds. The spatiotemporal distribution of seven fluorescent alkyne compounds was examined during 48 h after immersion (10 µM) or microinjection (2 mg/kg) in the pericardial cavity (PC), intraperitoneally (IP) and yolk sac (IY) of 3 dpf zebrafish eleuthero-embryos. By modelling the fluorescence of whole-body contours present in fluorescence images, the main pharmacokinetic (PK) parameter values of the compounds were determined. It was demonstrated that especially in case of short incubations (1–3 h) immersion can result in limited intrabody exposure to compounds. In this case, PC and IP microinjections represent excellent alternatives. Significantly, IY microinjections did not result in a suitable intrabody distribution of the compounds. Performing a QSPkR (quantitative structure-pharmacokinetic relationship) analysis, LogD was identified as the only molecular descriptor that explains the final uptake of the selected compounds. It was also shown that combined administration of compounds (immersion and microinjection) provides a more stable intrabody exposure, at least in case of a prolonged immersion and compounds with LogD value > 1. These results will help reduce the risk of false negative results and can offer an invaluable input for future translational research and safety assessment applications.

## Introduction

Zebrafish (*Danio rerio*) is a small vertebrate that has gained increasing popularity not only as an animal model for translational research but also to assess the toxicity of compounds such as drug leads, cosmetics, foods, and environmental samples^1–3^. The key advantages of using this animal model include its high genetic, physiologic, and pharmacologic homology with humans, its small size, high fecundity rate, rapid development, and semi-transparent appearance during the larval stages^4,5^. Particularly, the semi-transparent appearance in combination with the *ex-utero* development has made it possible to screen for developmental effects after compound exposure using nothing more than a low magnification microscope. Ever since transgenic technology has become widely established and zebrafish with fluorescent highlighted organs could be generated^2^, more detailed screens for organ specific toxicities such as hepato-^6–8^, nephro-^9^ cardio-^8^^,^^10^, and neurotoxicity^8,11^ using fluorescence microscopy has emerged. In addition, it has been established that zebrafish can determine toxicity of pharmaceuticals and chemicals in general, with a specificity of 89–90%, sensitivity of 68–80%, and an accuracy of 78%^8,12^. Zebrafish is thus filling a gap between the affordable, fast, but too simple in vitro models and the more sophisticated but costly and time-consuming murid studies^13^ as it combines the high through-put capacity of in vitro assays with the benefits of being an in vivo model.

However, zebrafish models also come with some limitations. One being that at least during the early stages of its development the metabolic capacity is limited^14^. Hence there is a risk for compounds to be identified as false negatives due to incomplete metabolism. Some methodological advances have been developed to address this issue. For instance, protocols have been established that allow zebrafish eleuthero-embryos to be exposed to compounds after prior in vitro metabolism by rat liver microsomes^15,16^.

Another limitation is the low uptake of compounds by zebrafish after immersion exposure, the most common administration route used in toxicity screens, possibly resulting in false negative outcomes^5,14,17,18^. The absorptive ability of zebrafish eleuthero-embryos and larvae is largely determined by the physicochemical properties of the compounds. Studies have shown that among a wider number of molecular descriptors, lipophilicity plays the largest role in absorption^19–21^. Although somewhat more time-consuming, a way to circumvent a possible relative lack of absorption, is to microinject compounds into the animals. The most commonly used injection site in zebrafish eleuthero-embryo is the yolk, consequently intra-yolk microinjections have also been automated^17,22^. Other routes to deliver compounds directly into larvae are intracardiac microinjections, as performed to evaluate the permeability of the blood–brain barrier by fluorescent compounds^23,24^, and intravenous microinjections to evaluate systemic infection of bacterial strains in the zebrafish^25,26^.

Despite the multiple and frequently used microinjection routes available, the disposition within the organism and rate of elimination of compounds injected in eleuthero-embryos and larvae has not been widely explored^14^. Hence it is not known to what extent microinjections typically result in a reliable exposure of internal organs and tissues to high concentrations of the compounds of interest. Therefore, not only a limited uptake of compounds during immersion, but also a limited body distribution or fast excretion of compounds after injection might unexpectedly lead to false-negative results. Moreover, a detailed comparison with results obtained after immersion using the same compounds is completely lacking.

In this study, we report on the absorption, intra-body distribution and elimination of seven fluorescent compounds in 3 dpf zebrafish eleuthero-embryos. The compounds were selected based on their different lipophilicity and delivered by immersion and microinjections in the pericardial, peritoneal cavity and yolk sac, respectively. By modelling the integrated fluorescence intensity of delineated whole-body contours present in the fluorescence images, we determined the main PK parameter values of the compounds. In addition, we examined whether a combined administration of compounds, i.e., immersion and microinjection, can offer an added value to the pharmacological activity or toxicity testing of compounds.

Our results will help to forecast the amount of chemical substance that is present in the zebrafish after administration via immersion, microinjection, or combined immersion/micro-injection, thus allowing a better-informed design of experiments and reducing the risk of false negative results.

## Results

### Calculation of molecular descriptors

We used SwissADME to compute a selection of molecular descriptors of seven fluorescent compounds, i.e., molecular weight (MW), polar surface area (TPSA), molar refractivity (MR) and number of H-bond acceptors (HBA), H-bond donors (HBD) and rotatable bonds (rotor). The results are presented in Table 1.

**Table 1:** Molecular descriptors of the fluorescent compounds (*in-silico* analysis using SwissADME) and the experimentally determined LogD values.

*Compound*	Sulfo-cyanine 3 alkyne (S-CY3A)	Sulfo-cyanine 5.5 alkyne (S-CY5.5A)	Sulfo-cyanine 5 alkyne (S-CY5A)	FAM alkyne, 5-isomer (FAMA)	Tamra alkyne 5-isomer (TAMRA)	Rhodamine 6G (R6G), 6-isomer (R6GA)	Cyanine3 alkyne (CY3A)
No	1	2	3	4	5	6	7
Structure							
MW G/MOL	691.9	1054.36	547.79	413.38	467.52	462.6	530.14
Rotor	13	18	11	3	6	7	10
HBA	7	13	1	6	4	2	1
HBD	1	1	0	3	1	1	1
MR	180.42	241.21	185.18	109.52	135.27	144.59	169.95
TPSA Å^2^	152.68	256.18	23.32	105.09	88.62	38.33	35.35
LogD	− 1.96	− 1.68	− 0.72	− 0.14	0.46	1.07	1.73

### Experimental determination of lipophilicity

In order to experimentally determine the lipophilicity (LogD) of the fluorescent compounds, we used the shake-flask method^46^. Briefly, 10 µM of compound was shaken in a mixture of two mobile phases, n-octanol and Danieau’s solution (eleuthero-embryo medium) and analysed with UHPLC. Compounds R6GA, FAMA and CY3A were separated by RPLC (Reversed Phase-Liquid Chromatography), whereas the least lipophilic compounds S-CY5.5A, TAMRA, S-CY3A and S-CY5 were analyzed by HILIC (Hydrophilic Interaction LC). Results show that the fluorescent compounds displayed LogD values in the range of − 1.92 to 1.73 (Table 1).

### Spatiotemporal imaging after immersion and microinjections

We then determined the spatiotemporal distribution of the fluorescent compounds. The eleuthero-embryos were exposed to the dyes by immersion or microinjection in the pericardial cavity (PC), intraperitoneally (IP) and in the yolk sac (IY) for 48 h starting from 3 dpf on, i.e. after hatching, thus avoiding the presence of the chorion that has been reported as a potential barrier for the absorption of compounds^21,27^.

The concentration (10 µM) and dose (0.5 ng, equivalent to 2 mg/kg) used were selected based on preliminary experiments and did not induce any sign of toxicity while transferring adequate and quantifiable fluorescence to the organism. At specific time periods after treatment, the eleuthero-embryos were immobilized by hypothermia and fluorescent microscope pictures were taken.

**Figure 1:**
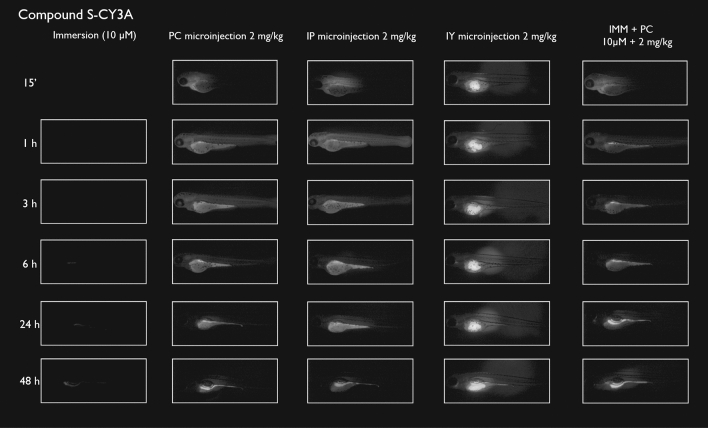
Representative pictures of the spatiotemporal distribution of the fluorescent compound S-CY3A. The eleuthero-embryos were exposed to the dye by immersion (10 µM) or microinjection (2 mg/kg) in the pericardial cavity (PC), intraperitoneally (IP) and in the yolk sac (IY), or by a combination of immersion and PC microinjection, for 48 h starting from 3 dpf. Selected images arranged in MS PowerPoint and GIMP (version 2.10.24 https://www.gimp.org/ 2021) Software.

**Figure 2:**
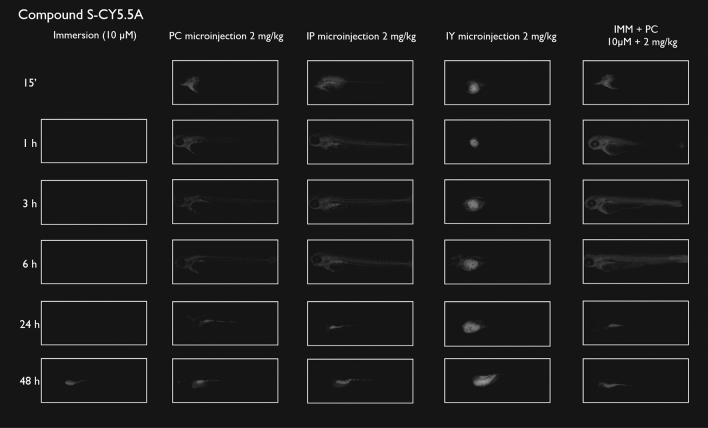
Representative pictures of the spatiotemporal distribution of the fluorescent compound S-CY5.5A. The eleuthero-embryos were exposed to the dye by immersion (10 µM) or microinjection (2 mg/kg) in the pericardial cavity (PC), intraperitoneally (IP) and in the yolk sac (IY), or by a combination of immersion and PC microinjection, for 48 h starting from 3 dpf. Selected images arranged in MS PowerPoint and GIMP (version 2.10.24 https://www.gimp.org/ 2021) Software.

**Figure 3:**
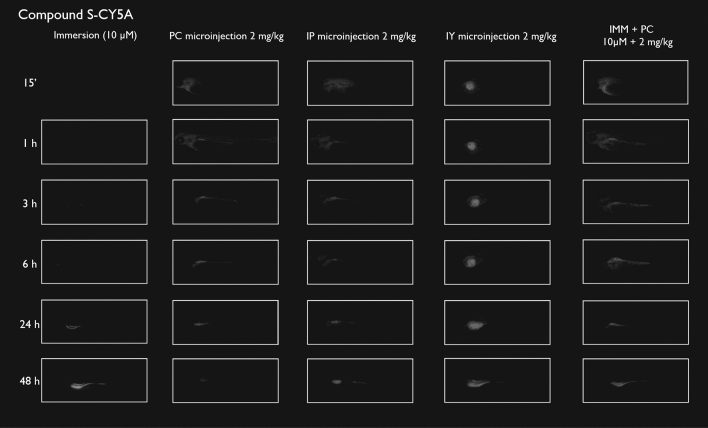
Representative pictures of the spatiotemporal distribution of the fluorescent compound S-CY5A. The eleuthero-embryos were exposed to the dye by immersion (10 µM) or microinjection (2 mg/kg) in the pericardial cavity (PC), intraperitoneally (IP) and in the yolk sac (IY), or by a combination of immersion and PC microinjection, for 48 h starting from 3 dpf. Selected images arranged in MS PowerPoint and GIMP (version 2.10.24 https://www.gimp.org/ 2021) Software.

**Figure 4:**
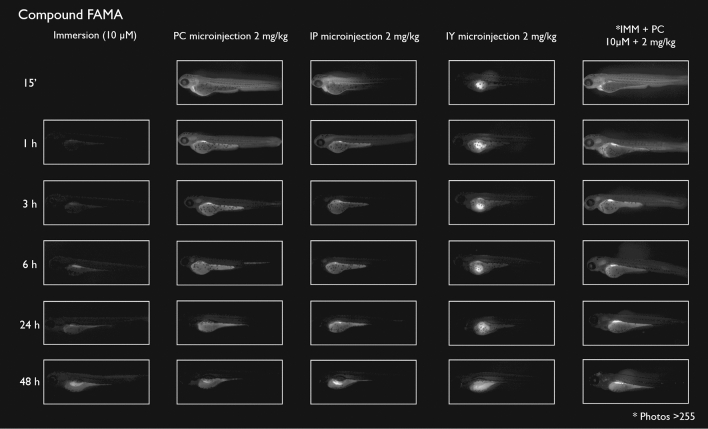
Representative pictures of the spatiotemporal distribution of the fluorescent compound FAMA. The eleuthero-embryos were exposed to the dye by immersion (10 µM) or microinjection (2 mg/kg) in the pericardial cavity (PC), intraperitoneally (IP) and in the yolk sac (IY), or by a combination of immersion and PC microinjection, for 48 h starting from 3 dpf. Selected images arranged in MS PowerPoint and GIMP (version 2.10.24 https://www.gimp.org/ 2021) Software.

**Figure 5:**
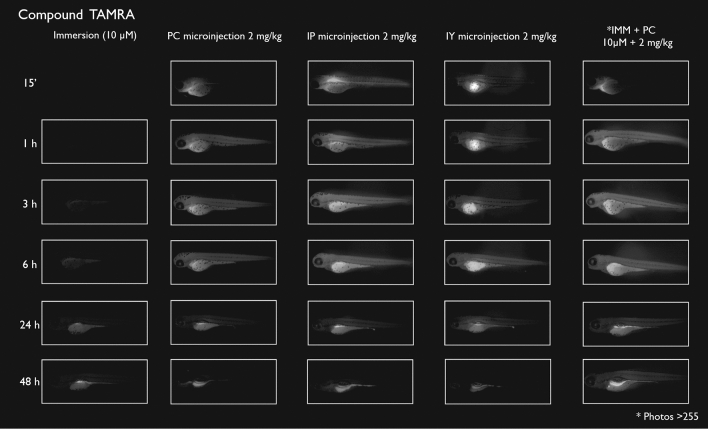
Representative pictures of the spatiotemporal distribution of the fluorescent compound TAMRA. The eleuthero-embryos were exposed to the dye by immersion (10 µM) or microinjection (2 mg/kg) in the pericardial cavity (PC), intraperitoneally (IP) and in the yolk sac (IY), or by a combination of immersion and PC microinjection, for 48 h starting from 3 dpf. Selected images arranged in MS PowerPoint and GIMP (version 2.10.24 https://www.gimp.org/ 2021) Software.

**Figure 6:**
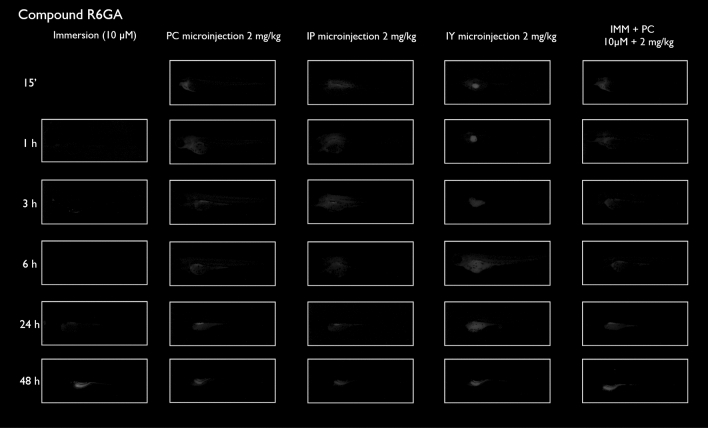
Representative pictures of the spatiotemporal distribution of the fluorescent compound R6GA. The eleuthero-embryos were exposed to the dye by immersion (10 µM) or microinjection (2 mg/kg) in the pericardial cavity (PC), intraperitoneally (IP) and in the yolk sac (IY), or by a combination of immersion and PC microinjection, for 48 h starting from 3 dpf. Selected images arranged in MS PowerPoint and GIMP (version 2.10.24 https://www.gimp.org/ 2021) Software.

**Figure 7:**
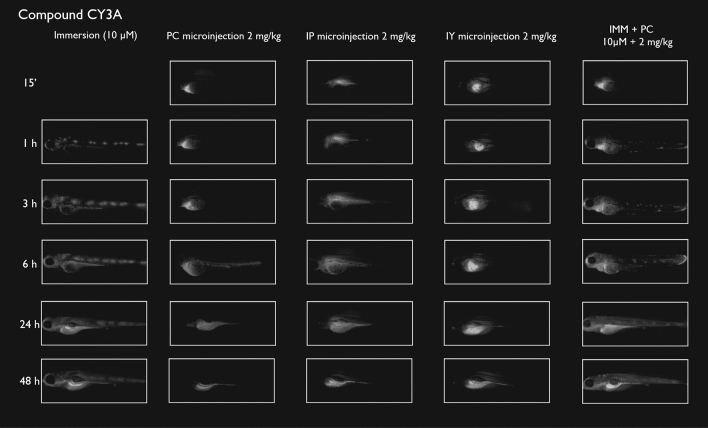
Representative pictures of the spatiotemporal distribution of the fluorescent compound CY3A. The eleuthero-embryos were exposed to the dye by immersion (10 µM) or microinjection (2 mg/kg) in the pericardial cavity (PC), intraperitoneally (IP) and in the yolk sac (IY), or by a combination of immersion and PC microinjection, for 48 h starting from 3 dpf. Staining of neuromast cells of the lateral line is visible 1 h post-immersion. Selected images arranged in MS PowerPoint and GIMP (version 2.10.24 https://www.gimp.org/ 2021) Software.

After immersion, four of the compounds i.e., S-CY3A, S-CY5.5A, S-CY5A and R6GA, were slowly taken up, especially during the first 6 h (Figs. 1, 2, 3, 6). The remaining three compounds, TAMRA, FAMA and CY3A (Figs. 4, 5, 7) were gradually taken up over time, with the fluorescent signal mainly localizing in the gastrointestinal system. CY3A was already clearly absorbed by the eleuthero-embryos after 1 h of immersion. Additionally, CY3A also presented fluorescence in the lateral line neuromast cells, starting from 1 h post exposure (Fig. 7).

Examining the intra-fish distribution after microinjections into the pericardial cavity, all fluorescent compounds distributed in the body of the eleuthero-embryo, hence providing good tissue exposure (Figs. 1, 2, 3, 4, 5, 6, 7). Specifically, S-CY3A (Fig. 1), S-CY5.5A (Fig. 2), S-CY5A (Fig. 3), FAMA (Fig. 4) and TAMRA (Fig. 5) rapidly distributed to the vasculature. After the signal reached the highest level, the total fluorescence dropped gradually, for some compounds faster than others, most probably by excretion via the cloaca. Intraperitoneal microinjections (Figs. 1, 2, 3, 4, 5, 6, 7) showed very similar results as obtained for the pericardial microinjections. Conversely, compounds injected in the yolk remained mainly localized at the microinjection site, except in case of TAMRA (Figs. 1, 2, 3, 4, 5, 6, 7).

As the uptake of compounds during immersion increases over time, whereas PC and IP injections result in the highest amount of compound present at an early time point, we wanted to investigate whether combining immersion (10 µM) and PC microinjection (2 mg/kg) would result in more stable intrabody levels of the fluorescent compounds. The images show that the level and intra-body fluorescence distribution as observed 48 h later was altered for compounds S-CY5A, TAMRA and CY3A as compared to the ones obtained for the PC microinjection route, whereas for all other compounds the visual differences were limited (Figs. 1, 2, 3, 4, 5, 6, 7).

### Quantification of whole-body fluorescence and PK analysis

We then quantified the relative amount of compound present in the zebrafish eleuthero-embryos by assessing the integrated fluorescence intensity of delineated whole-body contours present in the fluorescence images (Figs. 1, 2, 3, 4, 5, 6, 7). Fluorescent images obtained after IY microinjections were not analysed as in most cases no redistribution of the compound could be observed across the zebrafish eleuthero-embryos.

The total measured fluorescence was proportional to total amount of compound present in the animal, as described by Eq. (1). $RF{U}_{T}$ denotes the sum of fluorescence intensity in the overall image, $A$ denotes the amount of drug in the zebrafish, and $FLUOR$ is a constant denoting the compound-specific fluorescence quantum yield.

1 $$RF{U}_{T}=FLUOR*A$$

**Figure 8:**
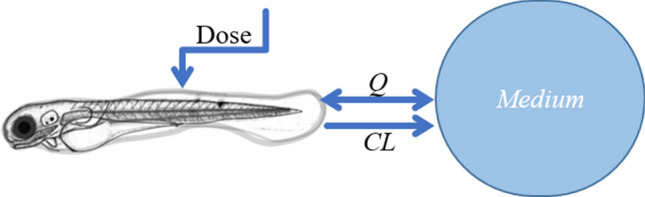
Schematic illustration of the 1-compartment model used to calculate PK parameters of the fluorescent compounds in the zebrafish eleuthero-embryo. One-way active clearance *CL* (L/h), passive exchange *Q* (L/h), compound in the medium (mg/L) and dose administrated by microinjection (mg/kg). The image was drawn using MS PowerPoint and GIMP (version 2.10.24 https://www.gimp.org/ 2021) Software.

To describe the disposition kinetics of each compound in the zebrafish, exploratory analysis of the fluorescence time profiles showed a fast distribution from the injection site for IP and PC injections, supporting a 1-compartment model as the most parsimonious description of the data. Exchange with the environment (at compound concentration M, in mg/L) was described as a superposition of one-way active clearance CL (L/h) and passive exchange Q (L/h) (Eqs. 2, 3 and 4, Fig. 8).

2 $$\frac{dA}{dt}=-CL\frac{A}{V}-Q\frac{A}{V}+QM$$

Simplifying by substitution of ${k}_{e}=\frac{Q+CL}{V}$:

3 $$\frac{dA}{dt}=-{k}_{e}*A+QM$$

Next, data fitting was performed with the following mathematical model:

4 $$A\left(t\right)=Dose*{e}^{-{k}_{e}t}+\frac{MQ}{{k}_{e}}(1-{e}^{-{k}_{e}t})$$

with Dose the injected dose (mg/kg), *M* the concentration in the medium (mg/L), *Q* the passive exchange with the medium (L/h) and ${k}_{e}$ the total elimination rate constant (h^−1^). When administering the compound through immersion, the injected dose is 0 mg/kg. When injecting the compound, the concentration in medium is 0. This model was fitted to all available data using non-linear regression in R version 4.0.3.

**Figure 9:**
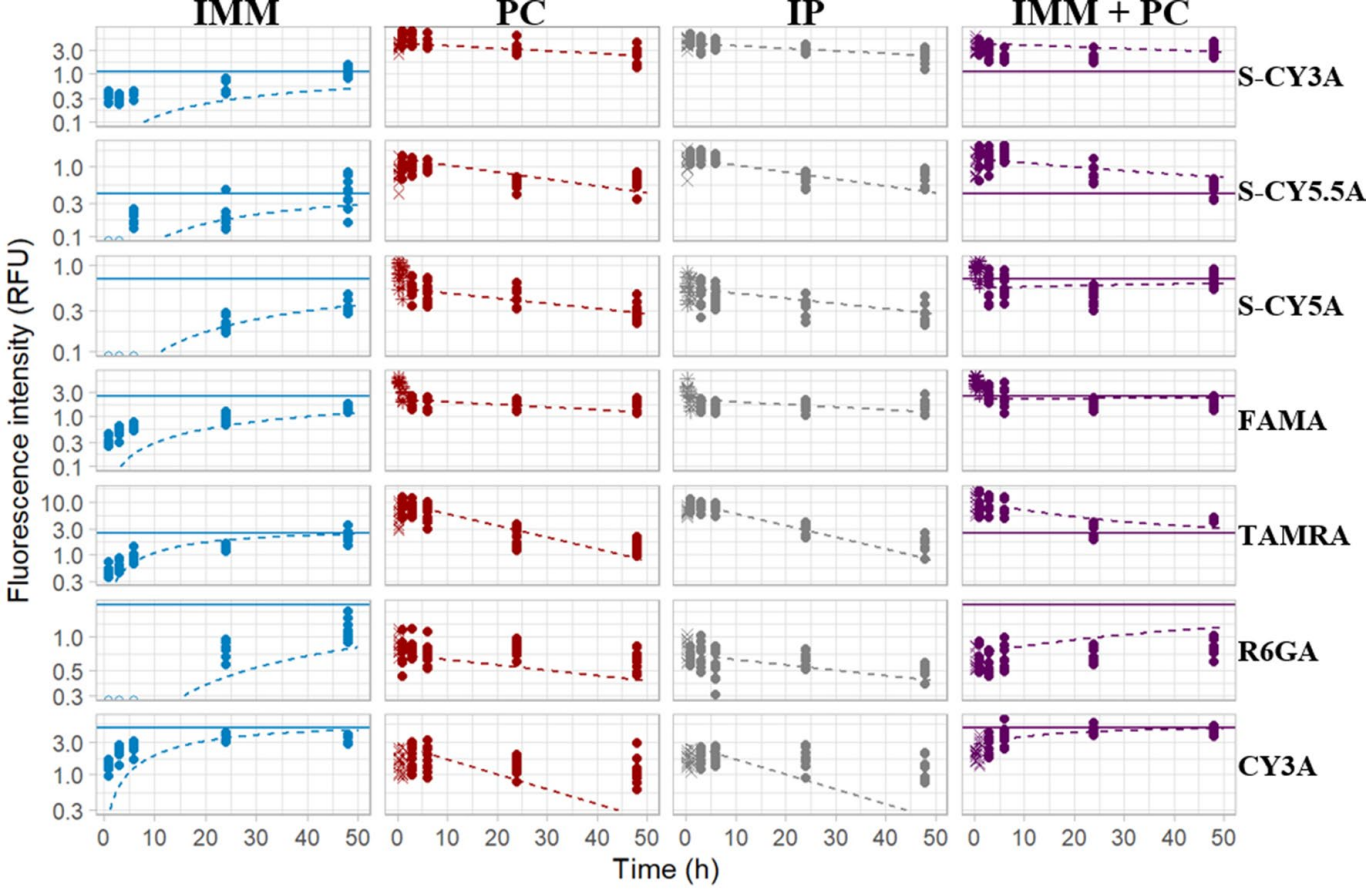
Fluorescence-time curves for all compounds and administration routes, with excluded data points marked as X symbols. Model prediction is presented as dotted line, model-predicted equilibrium fluorescence after immersion as solid horizontal line. The image was produced using R (version 4.0.3 https://www.r-project.org/ 2020) and colours arranged in GIMP (version 2.10.24 https://www.gimp.org/ 2021) Software.

Residual error plots were used to identify observations with poor fit. A high residual error implies these data are poorly described by the pharmacokinetic model, for some data points possibly due to fluorescence quenching. These observations were censored per compound and excluded from the modelling dataset (Fig. 9). The final model showed low residual error (Supplementary information, Figure S2) and low bias. Consequently, PK parameters like *k*_*e,*_ ${t}_\frac{1}{2}e$ (as calculated from ln(2)/*k*_e_) and *Q* could be accurately identified with low standard errors (Table 2).

**Table 2:** Values of fluorescence independent *PK* parameters calculated by modelled data, and residual standard error.

Compound	S-CY3A	S-CY5.5A	SCY5A	FAMA	TAMRA	R6GA	CY3A
*k*_*e*_ (h^−1^ × 10^−2^)	0.012	0.023	0.014	0.012	0.052	0.011	0.049
${t}_\frac{1}{2}$(h)	57.40	30.26	50.41	57.41	13.35	64.33	13.92
*Q* (L/h × 10^−9^)	0.23	0.34	1.61	1.71	1.45	3.23	8.37
Residual standard error	1.04	0.28	0.13	0.61	1.89	0.24	0.99

Compounds: S-CY3A (1), S-CY5.5A (2), S-CY5A (3), FAMA (4), TAMRA (5), R6GA (6) and CY3A (7). Confidence interval 95%.

**Figure 10:**
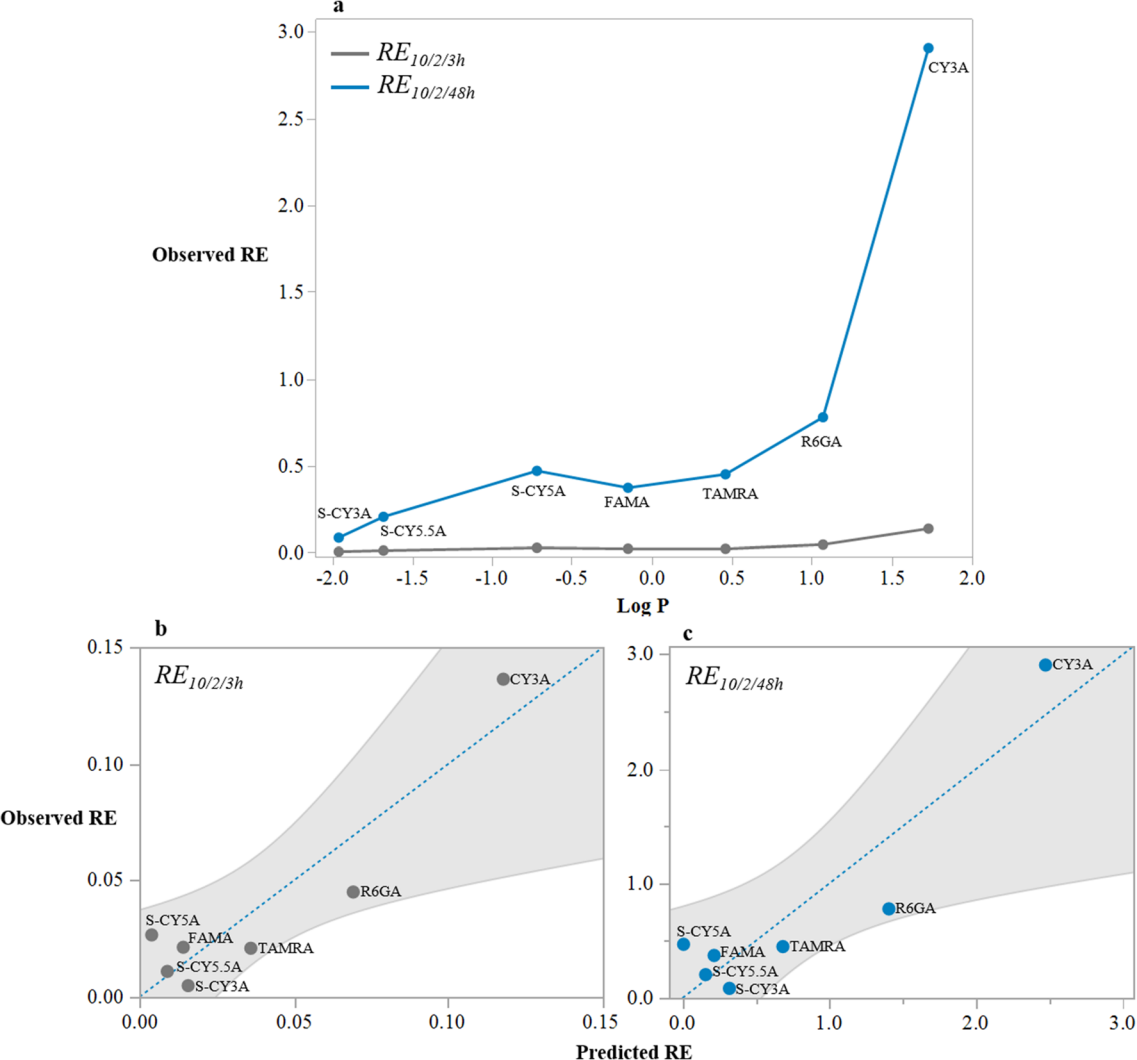
Relationship between observed *RE* and LogD for the short incubation (0–3 h) and prolonged incubation period (0–48 h) (**a**). (**b**) Plot of the observed *RE*_10/2_ versus the predicted *RE*_10/2_ in case of the short incubation (0–3 h), (**c**) and in case of the prolonged incubation period (0–48 h). Dashed line is the line of fit, shade color is the confidence interval of the model (95%). The image was produced using JMP (version 15.1. SAS Institute Inc., Cary, NC, 2019) and arranged in GIMP (version 2.10.24 https://www.gimp.org/ 2021) Software.

Moreover, we calculated the *AUC*_0–48h_ values based on fitted functions that represents the total compound exposure across time for the immersion, PC and IP microinjection and the combined treatment conditions. To define the Relative Exposure (*RE*_10/2_) of the compound after immersion at 10 µM, we calculated the ratio of *AUC*_0–48h_ immersion vs the *AUC*_0–48h_ PC microinjection (2 mg/kg) (Fig. 10a) as defined in Eq. (5):

5 $$RE_{{10/2/h}} = \left( {{{AUC_{{\text{Im} m}} } \mathord{\left/ {\vphantom {{AUC_{{\text{Im} m}} } {AUC_{{Inj}} }}} \right. \kern-\nulldelimiterspace} {AUC_{{Inj}} }}} \right)$$

In general, the results show that in case of short incubations (3 h) the relative exposure was low to very low (range: < 0.01–0.05) for all compounds, and somewhat higher for the most lipophilic compound CY3A (i.e., *RE*_10/2/3h_: 0.14) (Fig. 10a). Additionally, the *RE* values for the 48 h incubation (i.e., *RE*_10/2/48h_) were low for the two least lipophilic compounds S-CY3A and S-CY5.5A (i.e., 0.08 and 0.21, respectively), and high to very high for two most lipophilic compounds R6GA and CY3A (i.e., 0.78 and 2.90, respectively). Of interest, the RE values of the other compounds plateaued around 0.5. The data therefore indicate that the total body exposure to most compounds during the 0–48 h period after immersion were lower than after microinjections (both PC and IP), except for the compound with the highest LogD value (1.73) (Fig. 10a).

In addition, comparing the passive exchange with the medium (*Q*) of the compounds reveals that CY3A is taken up the fastest, whereas compound S-CY5.5A is the slowest one to be exchanged (Table 2). PC and IP microinjections resulted in similar *AUC* and elimination half-lives. Additionally, compounds FAMA and CY3A were the slowest to be eliminated from the fish.

As expected, for each one of the 7 compounds the AUC of the combination exposure, i.e. immersion + PC microinjection from 0 to 48 h, was higher than the AUC by microinjection alone (whether PC or IP) (Fig. 9). Thereby we confirmed that the combination of these administration routes results in an additive effect and continuous exposure to the compounds. Likewise, to evaluate the intrabody exposure as a result of that combined treatment, we calculated the relative *AUC* contribution (*RC*) of the immersion and PC exposure route as compared to *AUC* obtained after combination treatment, as described in Eq. (6).

6 $$RC_{h} = \left( {{{AUC_{{Exposureroute}} } \mathord{\left/ {\vphantom {{AUC_{{Exposureroute}} } {AUC_{{Combination}} }}} \right. \kern-\nulldelimiterspace} {AUC_{{Combination}} }}} \right) \times 100$$

**Figure 11:**
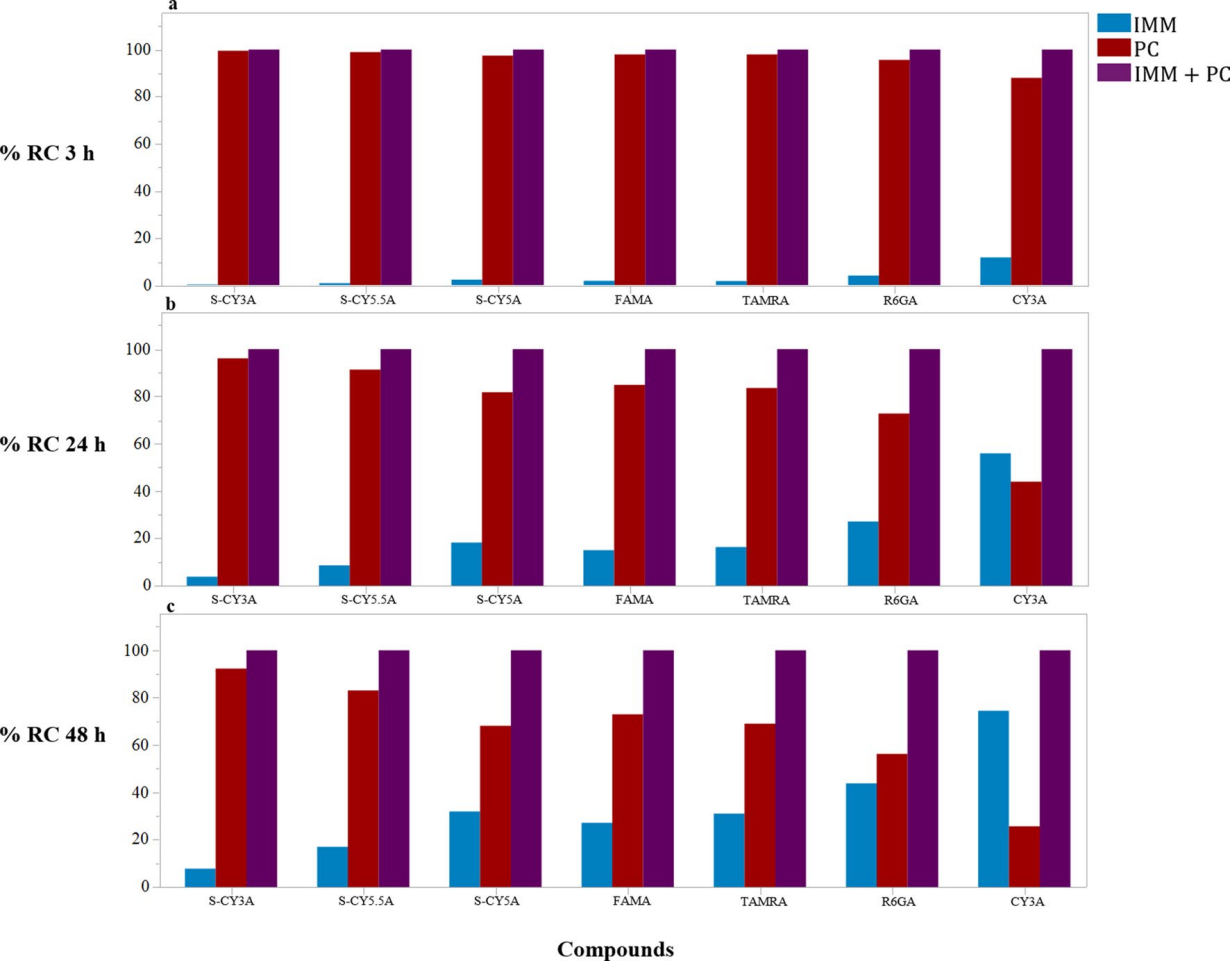
Histograms of Relative *AUC* contribution (*RC*_*h*_) of the immersion and PC exposure route as compared to *AUC* obtained after combination treatment for a 3 h treatment (**a**), 24 h treatment (**b**) and a 48 h treatment (**b**). The image was produced using JMP (version 15.1. SAS Institute Inc., Cary, NC, 2019) and arranged in GIMP (version 2.10.24 https://www.gimp.org/ 2021) Software.

The results for the 3 h-exposure (i.e., *RC*_3h_) (Fig. 11a) show that the intrabody exposure is mainly due to the microinjection route, and no effective additional effect resulted from the immersion route, except to a limited degree in case of the most lipophilic compound (CY3A). However, in case of a 24 h-exposure (i.e., *RC*_24h_) (Fig. 11b) and especially a 48 h-exposure (i.e., *RC*_48h_) (Fig. 11c), a limited to substantial contribution of the immersion route to the total intrabody exposure of the compound was demonstrated.

### QSPkR: quantitative structure-pharmacokinetic relationship analysis

To identify the molecular descriptors that best explain the calculated PK parameters (QSPkR quantitative structure-pharmacokinetic relationship), we first evaluated and managed the molecular descriptors in groups of no covariances prior to a multiple linear regression analysis (Supplementary information, Table S1).

Then we evaluated the QSPkR of the calculated rates (*k*_*e,*_${t}_\frac{1}{2}$_*,*_* Q*, and *RE*). Results show that the LogD values (− 1.96 to 1.73) present a parabolic relationship with *Q* (Table 3), *RE*_10/2/3h_ (Table 3), and *RE*_10/2/48h_ (Table 3) (R^2^ 0.817, RMSE 1.20e−09, P < 0.015; R^2^ 0.755, RMSE 0.483, P < 0.027; R^2^ 0.774, RMSE 0.022, P < 0.023; respectively). The stepwise multiple linear regression did not identify any statistically significant model for *k*_*e,*_ and ${t}_\frac{1}{2}e$. In Fig. 10 plots of the observed *RE*_10/2_ versus the predicted *RE*_10/2_ in case of short incubations (0–3 h) (Fig. 10b) and in case of the prolonged incubation period (0–48 h) (Fig. 10c) are shown.

**Table 3:** Multiple linear regression of PK parameters and molecular descriptors as explanatory variable.

PK Parameter	Model	R^2^ adj	RMSE	P value
*Q*	= 1.362e−9 + 1.696e−9 (LogD) + (LogD + 0.179)^2^ 8.409e−9	0.817	1.20e−09	0.015
*RE*_10/2/3h_	= 0.018 + 0.026 (LogD) + (LogD + 0.179)^2^ 0.015	0.774	0.022	0.023
*RE*_10/2/48h_	= 0.297 + 0.539 (LogD) + (LogD + 0.179)^2^ 0.341	0.755	0.483	0.027

Only statistically significant models are shown.

R^2^ adj: R^2^ adjusted. RMSE: root mean square error. P value (< 0.05).

## Discussion

In this study we examined the spatiotemporal distribution of seven photostable fluorescent small-molecules in zebrafish eleuthero-embryos and subsequently investigated their PK characteristics. All compounds were terminal alkyne derivatives that are completely inert biologically^28,29^, unlike many other commercially available fluorescent derivatives that contain a chemical bio-reactive linker. In this way, we guaranteed that the body and tissue distribution of the compounds was determined by their intrinsic chemical characteristics only, and not by their reactivity towards biomolecules like peptides and proteins. Moreover, since the metabolic capacity of zebrafish eleuthero-embryos is very limited^15,16^, a straightforward correlation can be assumed between the intrabody fluorescence observed and the amount of compound present. We initially selected compounds with cLogD values that varied from low to high lipophilicity (calculated by ChemDraw v18). Subsequently experimental LogD values were determined so that accurate values corresponding to the actual experimental conditions were used.

Spatiotemporal imaging after pericardial (PC) and intraperitoneal (IP) microinjections showed that compounds distributed rapidly over the entire body. PC and IP results were comparable showing that IP microinjections are a proper alternative to pericardial exposure in the zebrafish eleuthero-embryo. This is an interesting outcome as PC microinjections become difficult to perform from 5 dpf on, whereas IP microinjections in zebrafish larvae can easily be performed even in juveniles and adult zebrafish^30,31^. Moreover, the IP exposure route represents an enterohepatic distribution which provides a similar distribution to the *per os* administration of medicines in humans^32^.

Significantly, IY microinjections did not result in a proper intrabody distribution of the compounds. This is somewhat unexpected as the IY exposure route is often used to deliver DNA and morpholino’s, and infect (eleuthero-)embryos with viruses, bacterial and cancer cells, especially since the technique is easy to perform^17,22,33–35^. However, in case of small chemicals, it appears that the molecules can become entrapped in the yolk that represents a dense amphiphilic environment consisting largely of cholesterol (40% of total lipid), phosphatidylcholine (17%), and phospholipo-glycoproteins (i.e., vitellogenins)^36^. In fact, it has been shown that yolk can selectively accumulate compounds from the surrounding aquatic environment, both by passive and active transport, involving yolk sac epithelium receptors in the latter case^37^.

We therefore show evidence that IY injections should be avoided when screening small compounds for toxic or pharmacological effects, as this route possibly results in a disproportionate number of false negative outcomes. Clearly, the outcome does not preclude the possibility of an effective compound transfer from the yolk to body tissues when IY injections are performed during a very early embryonal stage (i.e., 0–3 hpf) as compared to the time point used in this study (i.e., 3 dpf). However, this issue is beyond the scope of the present study and requires further systematic characterization of the body distribution of IY-injected fluorescent dyes as a function of embryonal development.

Next, we quantified the integrated fluorescence intensity of delineated whole-body contours of the eleuthero-embryos after immersion and after the PC and IP microinjections, and modelled the data using a one-compartmental PK model. Notably, in case of some microinjected compounds, the fluorescent signal was not maximal immediately upon injection, likely as the result of quenching associated with self-assembled aggregates of the compounds present in high concentrations at the injection spot^38^. As a consequence, we modelled the microinjection data between 1 h or 3–48 h, censored per compound. The outcome demonstrates the feasibility to extract PK data from time-dependent series of 2D-fluomicrographs. Furthermore, the quantitative results concerning the PC and IP microinjections also confirm that the two routes result in a similar PK behavior.

Noteworthy, when comparing bio-characteristics of fluorescent compounds, it should be borne in mind that quantitative results obtained are critically dependent on the fluorescence quantum yield of the individual compounds and the equipment settings used. Hence, absolute fluorescence intensities of different compounds (e.g., as in case of *AUC* data) cannot be mutually compared. However, to identify and correlate the PK behavior of the compounds, we calculated their Relative Exposure values (*RE*_10/2_) both after a 3 h- (short exposure) and a 48 h-period (prolonged exposure), as well as their *k*_e_, (*t*$\frac{1}{2}$)*e*, and *Q* that all are fluorescence-independent.

For obvious reasons, the *RE* values critically hinge on the concentration (10 µM), the dose (2 mg/kg) and the exposure time used. Zebrafish eleuthero-embryos and larvae can be exposed to higher concentrations of chemicals (up to mM range)^18^, often depending on the maximum water-solubility of the compounds, and consequently one would expect correspondingly higher intra-body concentrations. However, in this study we selected a rather low but biologically relevant concentration and dose, as often used in preclinical experiments and zebrafish studies^8,39–41^. We found that the *AUCs* obtained for both the immersion and microinjections conditions were of the same order of magnitude, and this for a large range of LogD values, at least after prolonged exposure (48 h). Importantly, our data also reveal that short immersions (1–3 h) that are frequently used when testing the pharmacological activity or toxicity of compounds, would typically underexpose intrabody tissues and organs to the test compound as compared to the outcome observed with a 2 mg/kg microinjection.

We further identified Relative Exposure values (*RE*_10/2/h_) and the passive exchange with the medium (L/h) (*Q*) as useful predictors of absorption by exploratory stages of model building. Among other molecular descriptors, i.e., polar surface area (TPSA), molar refractivity (MR), number of H-bond acceptors (HBA), H-bond donors (HBD) and rotatable bonds (rotor), the QSPkR analysis identified LogD as the most important physicochemical descriptor to explain the PK parameters. These findings align with studies on the absorption of compounds in zebrafish eleuthero-embryo^19–21^ that have found that the higher the lipophilicity, the higher the uptake.

On the other hand, we did not find any relationship with the other descriptors that have been defined by Long et al. as predictive parameters for the absorption of compounds in zebrafish^19^. Possibly, this is due to fact that only a limited number of compounds was used in our work, thereby underestimating the effect of physicochemical characteristics that are less dominantly influencing absorption processes. However, the interesting work by Long and collaborators^19^ is based on the functional (in)activity of compounds used at different concentrations, as reported in literature, whereas in this study the relative intra-body exposure was considered, using a single concentration and dose. Likely, these differences in computational and experimental methodology affected the outcome of both studies.

Additionally, we investigated whether a combined administration of compounds by immersion and pericardial microinjection offers an extra benefit, as a continued higher exposure of intrabody tissues and organs to compounds might reduce the risk to obtain false negative results during pharmacological or toxicity screens. The outcome clearly demonstrates that only in case of a prolonged immersion (i.e., 48 h) an additional intrabody exposure can be expected, especially for lipophilic compounds (i.e., LogD > 1). In case of short exposures (1–3 h) the contribution to the intrabody concentration of the compound after immersion is very limited as compared to the one reached after microinjection.

In conclusion, in this study we compared the disposition of fluorescent compounds within the body using different exposure routes frequently used in zebrafish eleuthero-embryos (3–5 dpf) at a specific and commonly used concentration and dose. Taken together, the data show that the immersion route can result in limited intrabody exposure to compounds, especially in case of short incubations (typically 1–3 h), possibly resulting in false-negative results in screening programs. In this case, PC and IP microinjections represent excellent alternatives. However, considering that often multiple thousands of compounds are tested in ZF drug screens, performing injections of compounds does not always seem feasible. Based on the results obtained in this study we recommend to employ prolonged incubation times (e.g. 24 h), at least in case compounds exhibit LogD values below 1. Alternatively, if not toxic to the eleuthero-embryos, higher concentrations than 10 µM can be used as well, although in the present study the relationship between immersion concentrations and relative uptake was not studied, and no final conclusions on this matter can be given.

We further demonstrated that the IY exposure route should be avoided and hence care needs to be taken when analyzing results from this type of exposure, even though it has been widely implemented and automated^17,22,34^. Finally, we also provide a mathematical model to predict the relative uptake of compounds as a function of time which can offer an invaluable input for future translational research and safety assessment applications.

## Material and methods

### Zebrafish care and maintenance

Adult AB zebrafish (*Danio rerio*) were reared at 28.5 °C on a 14/10 h light/dark cycle according to standard zebrafish aquaculture conditions^42^. Food was given to the adult fish ad libitum while minimizing the surplus. Depending on the developmental stage of the fish, live food (i.e., freshly hatched nauplia of *Artemia salina*) and dry food (commercial fish diet) was given. Eleuthero-embryos were collected from natural spawning and fostered in Danieau’s solution (17 mM NaCl, 0.2 mM KCl, 0.18 mM Ca(NO3)2, 0.12 mM MgSO4 and 1.5 mM HEPES buffer pH 7.1–7.3)^43^. All procedures were carried out according to the Declaration of Helsinki and conducted following the ARRIVE guidelines^44^ and the guidelines of the European Community Council Directive 2010/63/EU, implemented in 2020 by the Commission Implementing Decision (EU) 2020/569 and all the relevant ethical regulations from the Ethics Committee of the University of Leuven (Ethische Commissie van de KU Leuven, approval number ECD 027/2019) and from the Belgian Federal Department of Public Health, Food Safety and Environment (Federale Overheidsdienst Volksgezondheid, Veiligheid van de Voedselketen en Leefmilieu, approval number LA1210261).

### Fluorescent compounds and their physicochemical properties

Fluorescent compounds were initially selected to have a wide range of cLogD (calculated lipophilicity) as defined by the ChemDraw calculator. These were purchased from Lumiprobe (Hannover, Germany): alkyne cyanine-based dyes: S-CY3A (CAS Nº A13B0), S-CY5.5A (CAS Nº A73B0), S-CY5A (CAS Nº A33B0) CY3A (CAS Nº A10B0), and alkyne xanthene-based dyes: FAMA (CAS Nº A41B0), TAMRA (CAS Nº A71B0) and R6GA (CAS Nº A52B0). They were dissolved in DMSO (99.9%) and frozen as 10 mM stock solutions at − 20 °C. Molecular descriptors and properties of the fluorescent compounds were calculated by the SwissADME platform^45^.

### Determination of experimental lipophilicity (LogD_o/w_)

The lipophilicity of the fluorescent compounds was determined following the EPA guideline OPPTS 830.7550 Partition coefficient (shake flask method)^46^ using Danieau’s solution and n-octanol as the two immiscible phases. Analyses were performed using an Agilent Infinity 1290 UHPLC system (Agilent Technologies, Waldbronn, Germany) consisting of an autosampler, quaternary pump and DAD-detector, operated with Open Lab software (version C.01.10, Agilent Technologies). FAMA, R6GA and CY3A were separated in RPLC mode on an Acquity BEH C18 column (100 × 2.1 mm, dp = 1.7 µm) from Waters (Milford, MA, USA) at a flow rate of 0.4 mL/min. Gradient elution was performed starting at 3:5:92 (v/v) ACN: 200 mM ammonium formate (adjusted to pH 3 with formic acid):MilliQ water, and changed to 82:5:13 (v/v) ACN: 200 mM ammonium formate (pH 3): MilliQ water in 11.5 min. S-CY5.5A, TAMRA, S-CY3A and S-CY5A were separated in HILIC mode on an Acquity BEH HILIC column (100 × 2.1 mm, dp = 1.7 µm) from Waters (Milford, MA, USA) at a flow rate of 0.4 mL/min. Gradient elution was performed starting at 95:5 ACN: 200 mM ammonium formate (pH 3) and changed to 60:5:35 ACN:200 mM ammonium formate (pH 3):MilliQ water in 11 min. The injection volume for all analyses was 1 µL and columns were kept at room temperature. All compounds were detected and quantified at 390 nm. For the quantification of the compounds, calibration samples were made in 50:50 ACN:MilliQ water for the compounds analysed in RPLC and 95:5 ACN:MilliQ water for compounds analysed in HILIC. Five concentration levels (10, 5, 2.5, 1.25, 0.63 µM) were used for all compounds except for R6GA, TAMRA and S-CY3A, for which six concentrations levels were used (10, 5, 2.5, 1.25, 0.63, 0.32 µM), and S-CY5A, for which four concentration levels (10, 5, 2.5, 1.25 µM) were used. Each sample was injected three times. The variation (calculated as the relative standard deviation, %RSD) for 3 injections was always below 10% (for all considered concentrations) and R^2^ values of the calibration curves were all above 0.998. All calculations concerning the evaluation of the recorded data were made in MS Excel (Microsoft Corporation, Seattle, USA). Finally, the LogD value was obtained as described in Eq. (7).

7 $$Log{D_{octanol/Danieau's}} = Concentratio{n_{Octanol}}/Concentratio{n_{Danieau's}}$$

### Treatment of zebrafish eleuthero-embryos with the fluorescent compounds

#### Immersion

3 dpf zebrafish eleuthero-embryos wildtype AB (n = 10 per compound) randomly selected were immersed using 6-well-plates (5 mL per well) of Danieau’s medium with a final DMSO concentration of 0.1% (v/v), containing the compounds at a concentration of 10 µM. Vehicle-treated control eleuthero-embryos were exposed to Danieau’s medium supplemented with 0.1% v/v DMSO.

#### Microinjections

3 dpf zebrafish eleuthero-embryos wildtype AB (n = 10 per compound) randomly selected were immobilized by cooling to 4 ºC and positioned on 1% (w/v) agarose plates at room temperature. All compounds were microinjected using glass needles fitted to a micromanipulator (MM-33) connected to a gas pressure microinjector (Eppendorf Femtojet set - AG 22331 Hamburg). Glass capillaries (W/FIL 1.0MM 4 in TW 100F-4) were pulled (Sutter Instrument CO. Model P-87 Cat N B100-58-15 Filament: FB330B – FB320B) by using program 5 (Heat 829, Pull 158, Vel 100, Time 150). Needles were filled with compounds dissolved in vehicle (DMSO/saline (1:1)). Microinjections were performed into the pericardial cavity (PC)^47^, into the yolk sac (IY)^35^, or into the peritoneal cavity (IP) at a volume of 1 nL and a dose of 2 mg/kg. For the intraperitoneal microinjection, the eleuthero-embryos were positioned in lateral right recumbence under a dissecting microscope. By using a micromanipulator, the needle was inserted posteriorly and ventrolateral to the swim bladder in the peritoneal cavity. Afterwards the eleuthero-embryos were transferred to 6-well plates. Control eleuthero-embryos were exposed to vehicle only.

The bodyweight of 3 dpf eleuthero-embryos was determined by weighing three batches of 20 embryos using an analytical balance after removing excess water with blotting paper. The average weight per eleuthero-embryo was 0.25 mg (SD ± 0.02).

#### Combination of exposure routes immersion and PC

3 dpf zebrafish eleuthero-embryos wildtype AB (n = 10 per compound) randomly selected were immobilized and positioned on 1% (w/v) agarose plates. All compounds were microinjected using glass needles, as previously described into the PC cavity. Afterwards, the larvae were selected and transferred to a 6-well-plate, into its corresponding compound at 10 µM.

The thus-treated eleuthero-embryos were kept in the incubator at 28.5 °C (in darkness) and taken out shortly at defined time points (15’, 1 h, 3 h, 6 h, 24 h and 48 h) for image analysis.

### Image analysis

Eleuthero-embryos were immobilized by hypothermia, rinsed three times with Danieau’s medium, and positioned latero-lateral (right lateral recumbency) in a drop of agarose (0.1%). To acquire images, a Leica MZ10F fluorescent stereomicroscope with a 4.0 × planapochromatic objective (10447243) was used, equipped with a Digital Color Camera Leica DFC310 FX (Software LAS 4.13). Filter sets were GFP 10446222 in case of compound FAMA, dSRED 10447079 in case of S-CY3A, CY3A and TAMRA, and CY5 10446366 in case of S-CY5.5A and S-CY5A. After manual delineation of the whole body (WB) contours of the zebrafish eleuthero-embryos using MetaMorph (Microscopy Automation & Image Analysis Software V.7.8.00), the fluorescence in the selected area was quantified as integrated fluorescence intensity (RFU) that adds up all fluorescence intensity values of the individual pixels. In case of the combination treatment the same correction factor for exposure time from the camera setting was applied in the case of FAMA and TAMRA, to avoid saturation of the images (Supplementary information. Figure S1)^48^. The selected images shown in the Figs. 1, 2, 3, 4, 5, 6 and 7 were arranged using MS PowerPoint (Microsoft Corporation, Seattle, USA) and the open-source GIMP (version 2.10.24 www.gimp.org 2021) software. All images were treated in the same way, adjusted to the same size, and the same RGB to grayscale conversion processing.

### Pharmacokinetic modelling and QSPkR

The designed model was optimized by a non-linear least squares modelling, using Gauss–Newton algorithms under the method of iterative fashion^49^, with R version 4.0.3, using stats::nls^50^.

For the QSPkR we performed multiple linear regression analysis using JMP, Version 15.1. SAS Institute Inc., Cary, NC, 1989–2019. The most appropriate model was identified in a stepwise search optimizing AIC (forward and backwards). We evaluated the association among the PK parameters (*k*_*e,*_ ${t}_\frac{1}{2}e$, *Q*) and *RE* of the fluorescent compounds, with the experimental LogD value and some in silico values.

## Supplementary Information

**Supplementary Information.**

## Data Availability

The datasets generated during and/or analysed during the current study are available from the corresponding authors on reasonable request. All data generated or analysed during this study are included in this published article (and its Supplementary Information files).
