# Supplementary material for: Spatiotemporal imaging and pharmacokinetics of fluorescent compounds in zebrafish eleuthero-embryos after different routes of administration
Source: Sci Rep. 2021 Jun 9;11:12229. doi: 10.1038/s41598-021-91612-6 (PMC8190279; doi:10.1038/s41598-021-91612-6)
Supplement: Supplementary file 1 — Supplementary Information. [file 41598_2021_91612_MOESM1_ESM.pdf]

Supplementary information

Spatiotemporal imaging and pharmacokinetic of fluorescent compounds in zebrafish eleuthero-embryos after different routes of administration

Authors

Marlly Guarin[1], Ruben Faelens[4], Arianna Giusti[1], Noémie De Croze[2], Marc Léonard[2], Deirdre Cabooter[3], Pieter Annaert*[4], Peter de Witte*[1] and Annelii Ny[1]

Table S1. Pairwise correlation of molecular descriptors. Only parameters with a significant correlation (P < 0.05) are listed.

Molecular Descriptor	***Molecular Descriptor***	***P value***
HBA	MW	0.0249
TPSA	MW	0.0157
MR	MW	0.0035
Rotor	MW	0.0027
MR	Rotor	<0.0001
TPSA	HBA	<0.0001

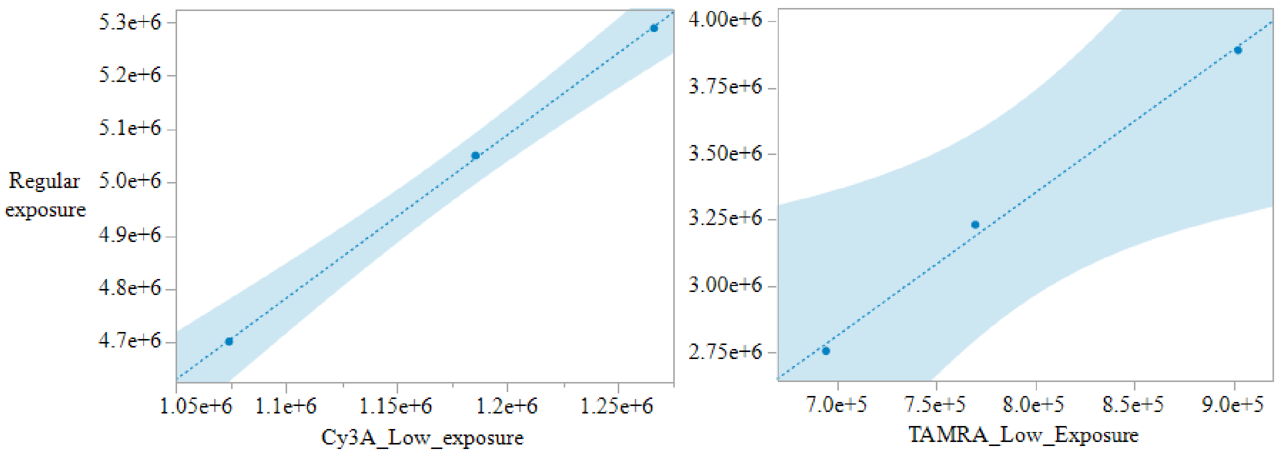

Figure S1. Linear regression of RFU collected at regular exposure time as used in experiments from the combination treatment *vs*. RFU collected at low exposure time. ***Left***. CY3A, R^2 0.999 P< 0.0099. ***Right.*** TAMRA, R^2 0.996 P< 0.0414. The image was produced using JMP (Version 15.1. SAS Institute Inc., Cary, NC, 2019) and arranged in GIMP (version 2.10.24 https://www.gimp.org/ 2021) Software.

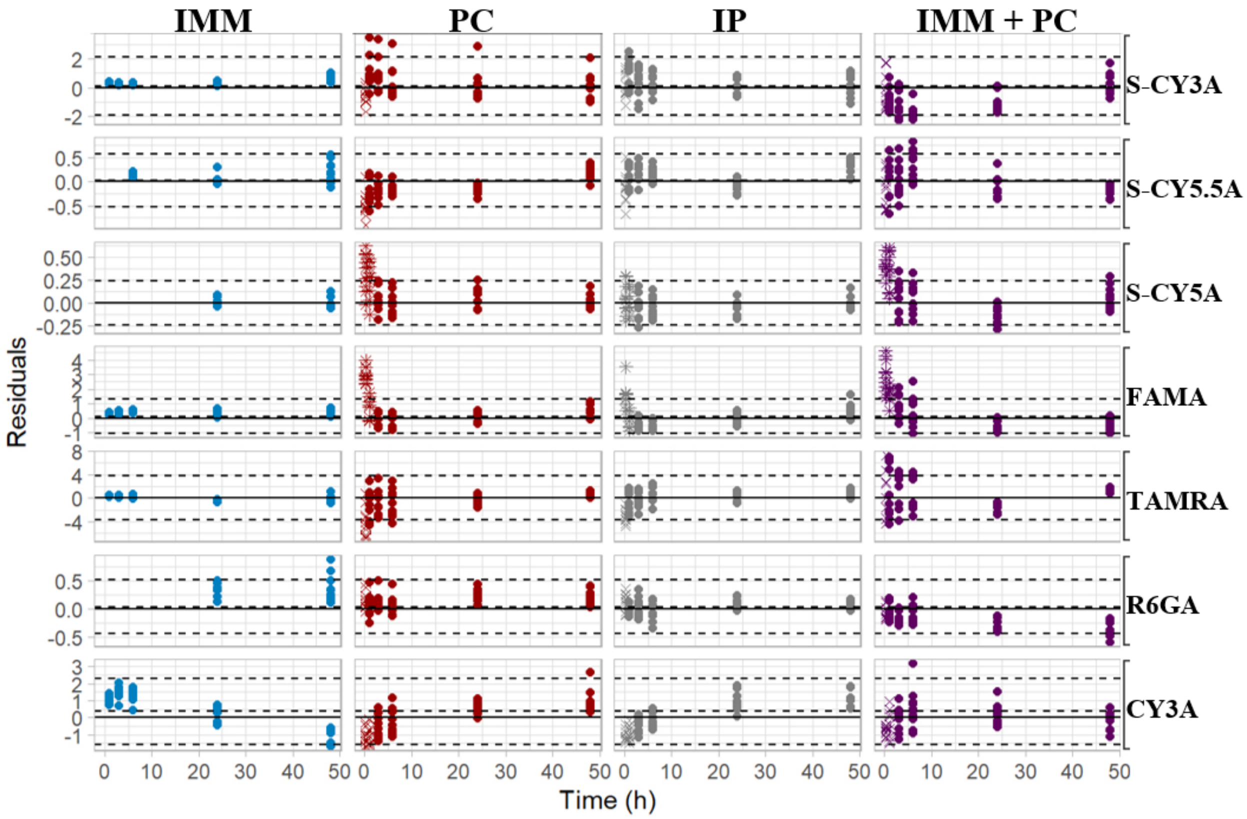

Figure S2. Standardized residuals after modelling data, excluding observations after microinjections (PC, IP) and combined treatment denoted by X at 0.25h, 1h and 3h, as censored per compound. The image was produced using R (version 4.0.3 https://www.r-project.org/ 2020) and arranged in GIMP (version 2.10.24 https://www.gimp.org/ 2021) Software.
